# The Role of Vitamin D in the Relationship Between Gender and Deep Vein Thrombosis Among Stroke Patients

**DOI:** 10.3389/fnut.2021.755883

**Published:** 2021-12-02

**Authors:** Jiejie Tao, Feiling Lou, Yuntao Liu

**Affiliations:** ^1^Department of Radiology, The First Affiliated Hospital of Wenzhou Medical University, Wenzhou, China; ^2^Department of Neurology, The First Affiliated Hospital of Wenzhou Medical University, Wenzhou, China

**Keywords:** stroke, deep venous thrombosis, gender, vitamin D, seniors

## Abstract

**Introduction:** Accumulating evidence had demonstrated that females had a higher risk of deep vein thrombosis (DVT) than males, but the mechanism was still unknown. Vitamin D was found to play an essential role in DVT, and gender may influence the serum vitamin D levels. This study aimed to explore whether vitamin D played a role in the gender difference in DVT.

**Materials and Methods:** A total of 444 patients with acute stroke were recruited, which were divided into the DVT group (*n* = 222) and the non-DVT group (*n* = 222). Serum vitamin D levels were measured after admission and were split into three categories, including deficiency (<50 nmol/L), insufficiency (52.5–72.5 nmol/L), and sufficiency (more than 75 nmol/L). Hierarchical regression analysis was adopted to analyze the relationship between gender and DVT, controlling the confounding factors.

**Results:** Females showed a higher proportion of DVT than males (60.7 vs. 42.5%, *p* < 0.001), and lower serum vitamin D levels than males (53.44 ± 16.45 vs. 69.43 ± 23.14, *p* < 0.001). Moreover, serum vitamin D levels were lower in the DVT group than in the non-DVT group (59.44 ± 19.61 vs. 66.24 ± 23.86, *p* < 0.001). Besides, the DVT group showed a lower proportion of vitamin D sufficiency than the non-DVT group (21.2 vs. 32.9%, *p* < 0.05). Hierarchical regression analysis showed that females had 2.083-fold (*p* < 0.001, unadjusted model) and 1.413-fold (*p* = 0.155, adjusted model) risk to develop DVT. In addition, the sufficiency status of vitamin D showed an independent protective effect on DVT (unadjusted model OR, 0.504, *p* = 0.004; adjusted model OR, 0.686, *p* = 0.011).

**Conclusion:** Females had a higher risk of DVT than males, and vitamin D may play an essential role in this relationship. Further studies are needed to explore whether vitamin D supplementation could reduce DVT risk in stroke patients, especially females.

## Introduction

Venous thromboembolic disease (VTE), comprising deep vein thrombosis (DVT) and pulmonary embolism (PE) (1, 2), affects nearly 1 per 1,000 individuals annually (3, 4) and has become a significant issue that threatens public health in China (5). DVT was prevalent in stroke patients, occurring in nearly 80% of those who did not receive preventive treatments (6). Without any prevention, DVT would happen in 2 to 7 days after stroke onset (7, 8).

The risk of DVT has been found to vary by gender and age. Females under 50 years old had a decreased incidence, whereas over 65 years old had a higher incidence of DVT (4). Moreover, previous studies in stroke patients also found that females had a higher risk of DVT than males (9, 10). The mechanisms underlying the increased risk of DVT in females may be as follows: pregnancy (11), hormone replacement therapy (12), estrogen antagonist therapy (13) and the antiphospholipid antibody syndrome (14), hormone loss during menopause (15), oral contraceptives (OCPs) (16). However, most of these factors were not common among stroke patients in China due to the differences such as age, culture, race, and disease (17). Therefore, further studies were needed to identify why female stroke patients had a higher risk of DVT.

Vitamin D is a fat-soluble vitamin involved in calcium and phosphorus metabolism to maintain bone health (18). The deficiency of vitamin D is a worldwide health issue (19, 20). Low vitamin D levels may increase the risk of stroke (21) and lead to a worse prognosis of stroke patients (22). It was worth noting that vitamin D was also crucial in developing DVT in stroke patients, and a small sample study showed that vitamin D deficiency showed a 4.683-fold risk for DVT (23). While the sample size of this study was small (*n* = 180), larger sample size was needed further to identify the relationship between vitamin D and DVT. Besides, previous research had also shown that vitamin D deficiency was more common in elderly and middle-aged females (24, 25), which may be owing to the factors such as gender difference in dietary intakes (26), less sunlight exposure (27), and estrogen loss (28).

Considering the gender difference in DVT and vitamin D levels and the role of vitamin D in DVT, this study assumed that lower vitamin D levels might be why female stroke patients had a higher risk of DVT.

## Materials and Methods

### Study Design

This research was a cross-sectional design. Patients were screened from March 2015–May 2020 in the First Affiliated Hospital of Wenzhou Medical University. The following were the criteria for inclusion: (1) ages over 40; (2) within seven days of the stroke onset; (3) stroke (ischemic or intracranial hemorrhage) validated by computed tomography (CT) and magnetic resonance imaging (MRI). The following were the criteria for exclusion: (1) Patients with a history of DVT; (2) Patients taking vitamin D, calcium supplements; (3) Patients with active cancer or other serious medical diseases. (4) Patients taking estrogen, OCPs, estrogen antagonists, or anticoagulant therapy before admission. (5) pregnancy. (6) antiphospholipid antibody syndrome.

Ultimately, 222 patients with DVT were admitted, and 222 age-matched patients without DVT were also admitted. This study was agreed by the Medical Ethics Committee of First Affiliated Hospital of Wenzhou Medical University and abide by the Declaration of Helsinki's principles.

### Data Collection

Data including demographic and clinical characteristics were collected. All blood samples were obtained between 6:00 and 8:00 a.m., after a night of fasting within 24 h of admission.

We selected serum 25-hydroxyvitamin D [25 (OH) D] as the indicator for vitamin D levels because it is the best indicator of its status (29). The fasting venous blood samples were obtained from patients within 24 h after admission and sent to the clinical laboratory within 4 h. The blood samples were centrifuged at 3,000 rpm for 10 min, and the supernatant was taken for further evaluation. The serum 25 (OH) D levels were measured by a competitive electrochemiluminescence protein binding assay (Cobas e602.Roche Diagnostics, Germany) in the lab of our hospital. The intraassay variation was between 7 and 10%, and the level over 75 nmol/l is considered vitamin D sufficiency in our hospital.

In the present study, vitamin D deficiency was specified as a 25 (OH) D of <20 ng/ml (50 nmol/L), insufficiency as 21–29 ng/ml (52.5–72.5 nmol/L), and sufficiency as more than 30 ng/ml (75 nmol/L) referred to the Endocrine Society's Practice Guidelines on vitamin D (30, 31).

### Diagnostic Criteria for DVT

Patients were examined using color Doppler ultrasonography (HDI 5,000 system with a 3–7 MHz linear array transducer, Philips ATL, Bothell, WA, USA) within seven days after admission.

### Statistical Analysis

Baseline demographic and clinical variables were compared between the groups. For a normal distribution test, the Kolmogorov-Smirnov test was used. The Mann-Whitney test was used for non-normally distributed variables, shown as the median (quartile). The normally distributed variables were represented by the mean and standard deviation (SD), analyzed by the student's *t*-test. Percentage and numbers were expressed for categorical variables and analyzed with chi-squared tests. The correlation analysis was conducted by the Pearson or Spearman rank analysis. Hierarchical regression analysis was further adopted to examine the relationship between gender and DVT, controlling the confounding factors. The confounding factors were categorized by accepted and validated classification criteria (such as WBC count, FIB, D-dimmer). Other variables such as PT, APTT were categorized according to the mean or median of the present values. SPSS 20.0 was used to conduct all analyses (IBM, SPSS, and Chicago, IL). Based on a two-sided test, *p* < 0.05 was regarded as statistically significant in all studies.

## Results

### Characteristics Between DVT and Non-DVT Groups

There was no statistical difference between the two groups regarding age, BMI, vital signs (except for body temperature), and stroke risk factors (all *p* > 0.05). In the DVT group, laboratory indicators including vitamin D levels, red blood cell (RBC), hemoglobin (HB), and Prothrombin Time (PT) were lower than the Non-DVT group (shown in Table 1, all *p* < 0.05). Padua score, the proportion of infection, fibrinogen (FIB), and D-dimmer levels were higher in the DVT group (shown in Table 1, all *p* < 0.05). Besides, RBC (*r* = 0.171, *p* < 0.001) and HB (*r* = 0.221, *p* < 0.001) were positively associated with vitamin D levels, other biochemistry markers such as WBC, PLT, PT, APTT, FIB, D-dimmer and EGFR were not related to vitamin D (all *p* > 0.05).

**Table 1:** Characteristics of all stroke patients.

**Characteristic**	**Total** **(*N* = 444)**	**Non-DVT group** **(*N* = 222)**	**DVT group** **(*N* = 222)**	**Statistic**	***P*-value**
**Demographic characteristics**					
Age (years), mean ± SD	70.80 ± 9.54	70.46 ± 9.67	71.13 ± 9.42	−0.736	0.462
Females, *n* (%)	183 (41.22)	72 (32.43)	111 (50.00)	13.423	<0.001^***^
BMI (kg/m2), mean ± SD	23.56 ± 2.53	23.49 ± 2.89	23.63 ± 2.12	−0.578	0.564
**Vital signs**					
Temperature (°C)	37.05 ± 1.04	36.90 ± 1.36	37.20 ± 0.52	−3.020	0.003^*^
Respiratory rate (breaths per min)	20.00 (19.00, 20.00)	20.00 (19.00, 20.00)	20.00 (19.00, 20.00)	2,4677.500	0.976
Heart rate (beats per min)	77.18 ± 14.73	76.26 ± 14.97	78.11 ± 14.46	−1.326	0.186
SBP (mmHg)	156.10 ± 24.04	156.92 ± 23.01	155.28 ± 25.05	0.718	0.473
DBP (mmHg)	84.84 ± 14.00	85.68 ± 14.17	83.99 ± 13.81	1.275	0.203
**Risk factors for stroke**, ***n*** **(%)**					
History of hypertension	307 (69.14)	152 (68.47)	155 (69.82)	0.042	0.837
History of diabetes mellitus	100 (22.52)	47 (21.17)	53 (23.87)	0.323	0.570
CAD	44 (9.91)	27 (12.16)	17 (7.66)	2.043	0.153
Current smoking	138 (31.08)	74 (33.33)	64 (28.83)	0.852	0.356
Current drinking	121 (27.25)	65 (29.28)	56 (25.23)	0.727	0.394
Atrial fibrillation	71 (15.99)	37 (16.67)	34 (15.32)	0.067	0.796
**Laboratory results**					
WBC (×10^9^/L), mean ± SD	7.88 ± 2.71	8.02 ± 2.86	7.74 ± 2.56	1.100	0.230
RBC (×10^12^/L), mean ± SD	4.37 ± 0.48	4.42 ± 0.49	4.33 ± 0.47	2.033	0.027^*^
HB (g/L), mean ± SD	133.70 ± 15.14	135.94 ± 13.78	131.46 ± 16.11	3.150	0.002^**^
PLT (×10^9^/L), mean ± SD	218.04 ± 69.54	217.43 ± 69.17	218.65 ± 70.06	−0.185	0.851
PT(s), mean ± SD	13.93 ± 1.43	14.06 ± 1.59	13.79 ± 1.23	2.006	0.046^*^
APTT(s), mean ± SD	37.42 ± 4.73	37.67 ± 4.63	37.18 ± 4.83	1.099	0.273
FIB(g/L), mean ± SD	3.51 ± 1.17	3.33 ± 1.09	3.70 ± 1.22	−3.335	0.001^**^
D-dimmer, median (IQR)	1.72 (0.19–3.25)	1.28 (0, 2.98)	2.24 (0.54–3.94)	19075	0.001^**^
EGFR (ml/ min/l.73m2), mean ± SD	83.76 ± 16.90	83.76 ± 15.78	83.77 ± 17.98	−0.099	0.921
Vitamin D (nmol/L), mean ± SD	62.84 ± 22.08	66.24 ± 23.86	59.44 ± 19.61	3.276	0.001^**^
**Clinical characteristics**					
Intracerebral hemorrhage (%)	153 (34.46)	80 (36.04)	73 (32.88)	0.359	0.485
NIHSS score, median (IQR)	5.00 (2.00, 10.00)	5.00 (2.00, 10.00)	6.00 (2.00, 10.00)	23852.500	0.558
Lower limb NIHSS score ≥ 2, median (%)	211 (47.55)	94 (42.30)	117 (52.70)	4.371	0.029^*^
NIHSS score at admission ≥ 11	92 (20.7)	45 (20.30)	47 (21.2)	0.055	0.815
Padua score, median (IQR)	4.00 (4.00, 5.00)	4.00 (3.25, 5.00)	4.00 (4.00, 5.00)	2,1498.000	0.015^*^
Infection (%)	71 (16.00)	45 (20.30)	26 (11.70)	6.052	0.014^*^

*DVT, deep vein thrombosis; BMI, body mass index; SBP, systolic pressure; DBP, diastolic pressure; CAD, Coronary Heart Disease; WBC, white blood cell count; RBC, red blood cell count; HB, hemoglobin value; PLT, platelet count; PT, Prothrombin Time; APTT, activated partial thromboplastin time; FIB, fibrinogen; EGFR, estimated glomerular filtration rate; NIHSS, National Institute of Health stroke scale; SD, standard deviation; IQR, interquartile*.

*** *p < 0.001*,

** *p < 0.01*,

* *p < 0.05*.

*Values were presented as the mean ± standard deviation (SD) or median (median± interquartile range, IQR) for continuous variables, and categorical variables were indicated as numbers (percentages). Student t-tests and Mann-Whitney U-tests were used according to data distribution. Chi-squared tests were used for categorical variables*.

The DVT group showed a higher percentage of females than the non-DVT group [(50.00%) vs. (32.43%), *p* < 0.001] (shown in Table 1). Patients with vitamin D sufficiency in the DVT group were fewer than the non-DVT [(21.2%) vs. (32.9%), *p* < 0.05]. In contrast, the proportion of insufficiency [(45.5%) vs. (35.6%), *p* < 0.05] and deficiency [(33.3%) vs. (31.5%), *p* > 0.05] were higher in the DVT group (though deficiency group did not reach statistically significance) (shown in **Table 4**).

### Correlation Between Vitamin D and Other Risk Factors

**Figure 1:**
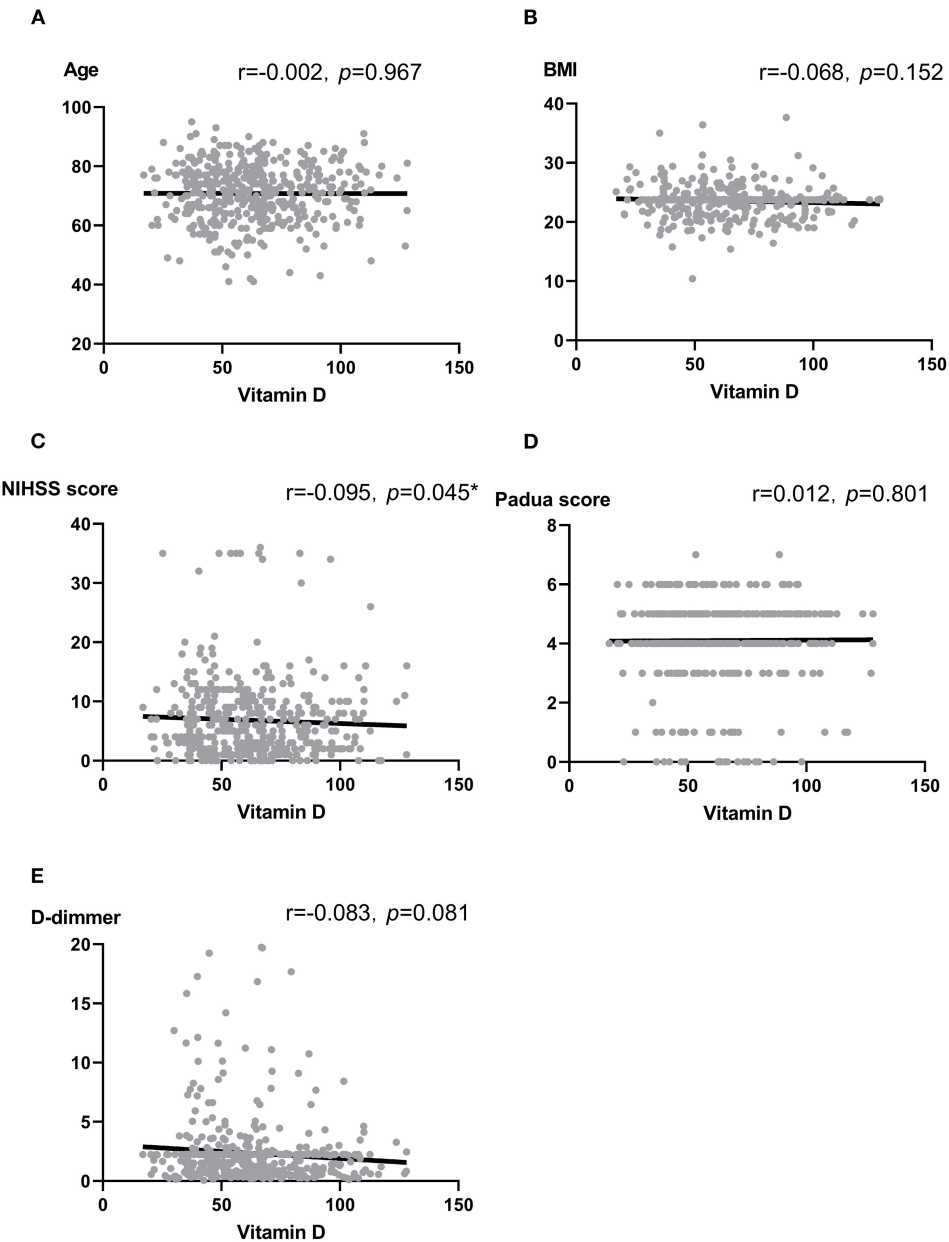
**(A–E)** Scatter plots: Association between vitamin D and Age **(A)**, BMI **(B)**, NIHSS score **(C)**, Padua score **(D)** as well as D-dimmer **(E)**. Note: r, correlation coefficient; BMI, body mass index; NIHSS, National Institute of Health stroke scale. Pearson correlation and Spearman analysis were used to evaluate the correlation according to the type of data. *p < 0.05.

We analyzed the correlation between vitamin D and some thrombotic risk factors reported in stroke patients (17, 32). Vitamin D was not associated with age, BMI, Padua score, and D-dimmer (all *p* > 0.05). In contrast, there was a marginally significant association between vitamin D and National Institute of Health stroke scale (NIHSS) score at the admission (*r* = −0.095, *p* = 0.045) (Figure 1).

### Characteristics Between Male and Female

**Figure 2:**
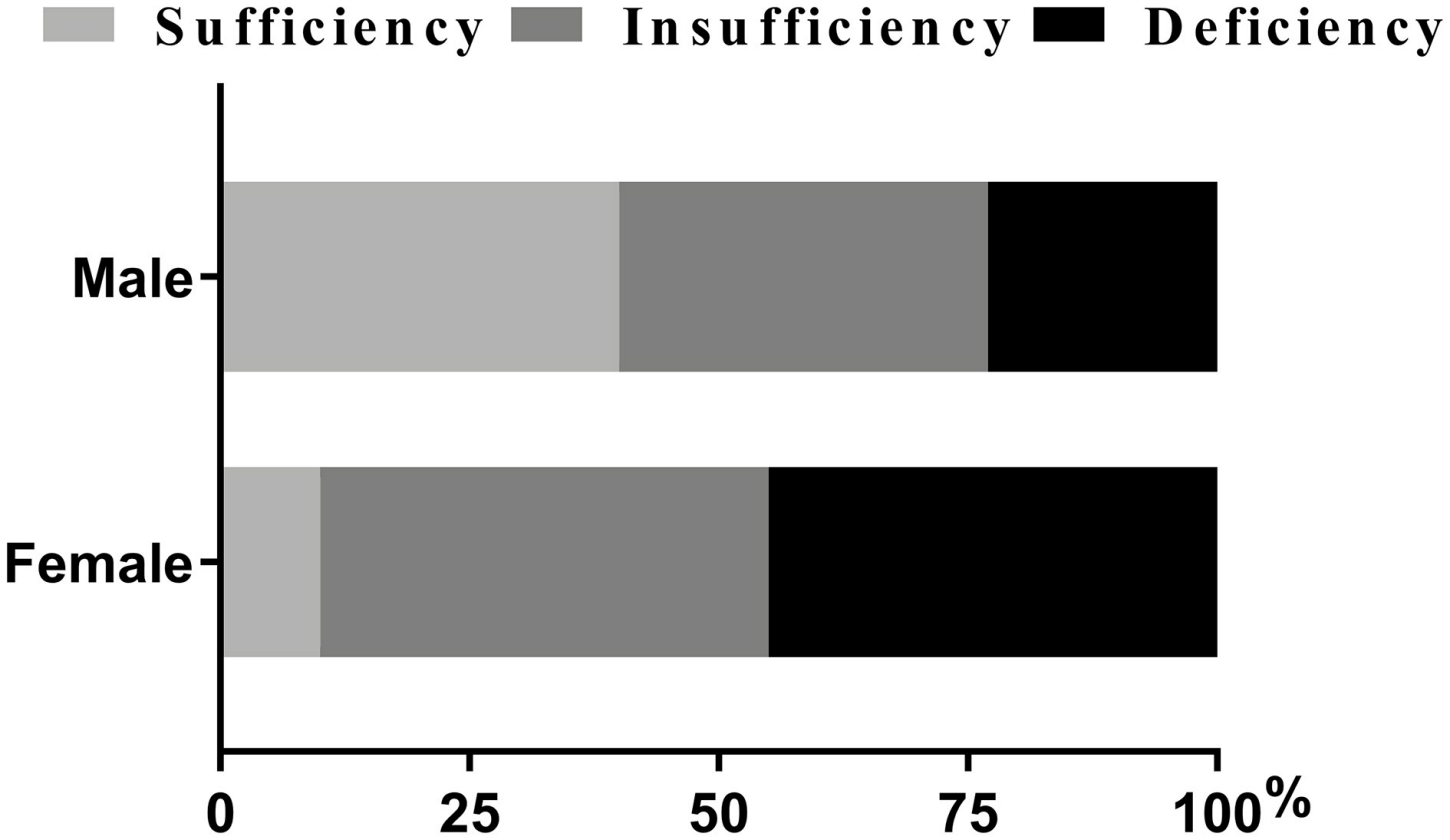
Percentage of patients with vitamin D status (%). Chi-squared tests and *post hoc* Bonferroni corrections for multiple comparisons were used for comparison between genders. Our data showed there were statistical differences in insufficiency and deficiency groups when categorized by genders.

As was shown in Table 2, variables such as temperature and heart rate were higher in females (all *p* < 0.01). Moreover, blood biomarkers of the females, such as APTT levels, were lower while PLT and D-dimmer levels were higher than the males (all *p* < 0.05). The proportions of NIHSS score ≥11 and lower limb NIHSS score ≥2 were also higher among the females (*p* < 0.01) (Table 2). It was worth noting that females showed lower levels of vitamin D and a higher proportion of DVT than the males (all *p* < 0.001) (Table 2). Besides, females had a higher proportion of deficiency [(45.40%) vs. (23.40%), *p* < 0.05] and insufficiency of Vitamin D status [(45.40%) vs. (37.20%), *p* > 0.05] than males, and had a lower proportion of sufficiency [(9.30%) vs. (39.50), *p* < 0.05] (Figure 2; Table 2).

**Table 2:** Characteristics of the patients categorized by gender.

**Characteristic**	**Total** **(*N* = 444)**	**Female group** **(*N* = 183)**	**Male group** **(*N* = 261)**	**Statistic**	***P*-value**
**Demographic characteristics**					
Age (years), mean ± SD	70.80 ± 9.54	70.97 ± 8.93	70.67 ± 9.96	0.33	0.741
BMI (kg/m2), mean ± SD	23.56 ± 2.53	23.60 ± 2.48	23.53 ± 2.58	0.258	0.796
**Vital signs**					
Temperature (°C)	37.05 ± 1.04	37.20 ± 0.45	36.94 ± 1.29	2.956	0.003^**^
Respiratory rate (breaths per min)	20.00 (19.00, 20.00)	20.00 (19.00, 20.00)	20.00 (19.00, 20.00)	2,4715	0.471
Heart rate (beats per min)	77.18 ± 14.73	80.52 ± 15.43	74.84 ± 13.77	3.994	<0.001^***^
SBP (mmHg)	156.10 ± 24.04	155.66 ± 24.99	156.41 ± 23.40	−0.321	0.749
DBP (mmHg)	84.84 ± 14.00	83.97 ± 14.19	85.45 ± 13.86	−1.093	0.275
**Risk factors for stroke**, ***n*** **(%)**					
History of hypertension	307 (69.14)	129 (70.49)	178 (68.20)	0.168	0.681
History of diabetes mellitus	100 (22.52)	43 (23.50)	57 (21.84)	0.088	0.767
CAD	44 (9.91)	25 (13.66)	19 (7.28)	4.218	0.04
Atrial fibrillation	71 (15.99)	36 (19.67)	35 (13.41)	2.691	0.101
**Laboratory results**					
WBC (×10^9^/L), mean ± SD	7.88 ± 2.71	7.65 ± 2.61	8.04 ± 2.78	−1.505	0.133
PLT (×10^9^/L), mean ± SD	218.04 ± 69.54	232.11 ± 82.93	208.18 ± 56.47	3.391	<0.001^***^
PT(s), mean ± SD	13.93 ± 1.43	13.86 ± 1.76	13.97 ± 1.13	−0.746	0.456
APTT(s), mean ± SD	37.42 ± 4.73	36.51 ± 4.84	38.06 ± 4.55	−3.404	<0.001^***^
FIB (g/L), mean ± SD	3.51 ± 1.17	3.56 ± 1.04	3.48 ± 1.25	0.685	0.494
D-dimmer (mg/L), median (IQR)	1.72 (0.19,3.25)	2.24 (0.46, 4.02)	1.44 (0,3.06)	20,809	0.02^*^
EGFR (ml/min/l.73m2), mean ± SD	83.76 ± 16.90	84.95 ± 15.01	82.93 ± 18.08	1.279	0.202
Vitamin D (nmol/L), mean ± SD	62.84 ± 22.08	53.44 ± 16.45	69.43 ± 23.14	−8.511	<0.001^***^
**Vitamin D status**				54.049	<0.001^***^
Sufficiency, *n* (%)	120 (27.00)	17 (9.30)	103 (39.50)		<0.05^*^
Insufficiency, *n* (%)	180 (40.50)	83 (45.40)	97 (37.20)		>0.05
Deficiency, *n* (%)	144 (32.40)	83 (45.40)	61 (23.40)		<0.05^*^
**Clinical characteristics**					
Intracerebral hemorrhage (%)	153 (34.46)	56 (30.60)	97 (37.16)	1.772	0.183
NIHSS score, median (IQR)	5.00 (2.00, 10.00)	6.00 (2.00, 11.00)	5.00 (2.00, 9.00)	25,526	0.215
Lower limb NIHSS score ≥ 2, median (%)	211 (47.50)	106 (40.60)	105 (57.4)	12.123	<0.001^***^
NIHSS score at admission ≥ 11	92 (20.7)	49 (28.60)	43 (16.50)	6.949	0.008^**^
Padua score, median (IQR)	4.00 (4.00, 5.00)	4.00 (4.00, 5.00)	4.00 (4.00, 5.00)	25,804	0.129
Infection (%)	71 (16.00)	32 (17.50)	39 (14.90)	0.518	0.472
The proportion of IDDVT (%)	222 (50.00)	111 (60.70)	111 (42.50)	14.139	<0.001^***^

*DVT, deep vein thrombosis; BMI, body mass index; CAD, Coronary Heart Disease; WBC, white blood cell count; RBC, red blood cell count; HB, hemoglobin value; PLT, platelet count; PT, Prothrombin Time; APTT, activated partial thromboplastin time; FIB, fibrinogen; EGFR, estimated glomerular filtration rate; NIHSS, National Institute of Health stroke scale; SD, standard deviation; IQR, interquartile*.

*** *p < 0.001*,

** *p < 0.01*,

* *p < 0.05*.

*Values were presented as the mean ± standard deviation (SD) or median (median± interquartile range, IQR) for continuous variables, and categorical variables were indicated as numbers (percentages). Student t-tests and Mann-Whitney U-tests were used according to data distribution. Chi-squared tests were used for categorical variables (Bonferroni correction was used if multiple comparisons)*.

### Gender, Vitamin D and DVT

We used hierarchical regression analysis to examine the role of vitamin D in gender and DVT (Tables 3, 4). Firstly, univariate binary regression suggested that female gender, infection, lower limb NIHSS score, Padua score, HB, PT, FIB, D-dimmer, and vitamin D status was found to be correlated with the presence of DVT (all *p* < 0.05) (Table 4). Then, multivariate-adjusted regression was conducted. In Model 1–3 (Table 3), the association between gender and DVT remained significant after adjusting the confounding factors (all *p* < 0.05) (Table 3). However, when categorized vitamin D status was added in model 4, the association between gender and DVT became insignificant (*p* = 0.155). The established model was shown in Table 5. Moreover, we also examined the interaction effect of DVT and gender on vitamin D levels using Two-way ANOVA analysis. However, the interaction effect was insignificant (*F* = 1.430, *p* = 0.232).

**Table 3:** Binary logistic models to examine the effect of gender on DVT.

	**OR (95%CI)**	***P*-value**
**Female**		
Unadjusted model	2.083 (1.418–3.062)	<0.001^***^
Model 1	2.069 (1.401–3.055)	<0.001^***^
Model 2	2.055 (1.377–3.068)	<0.001^**^
Model 3	1.582 (1.010–2.479)	0.045^*^
Model 4	1.413 (0.878–2.273)	0.155

*Take insufficiency of vitamin D group as the reference*.

*Model 1 included age ≥ 65 years, temperature ≥ 38°C, intracerebral hemorrhage, and infection*.

*Model 2 included model 1 and added NIHSS score at admission ≥ 11, Lower limb NIHSS score ≥ 2, BMI ≥ 25, and Padua score ≥ 4*.

*Model 3 included model 2 and added RBC (categorized), HB (categorized), PLT (categorized), PT (categorized), APTT (categorized), FIB (categorized) and D-dimmer (categorized)*.

*Model 4 included model 3 and added vitamin D (categorized)*.

*DVT, deep vein thrombosis; BMI, body mass index; CAD, Coronary Heart Disease; WBC, white blood cell count; RBC, red blood cell count; HB, hemoglobin value; PLT, platelet count; PT, Prothrombin Time; APTT, activated partial thromboplastin time; FIB, fibrinogen; EGFR, estimated glomerular filtration rate; NIHSS, National Institute of Health stroke scale; OR, odds ratios; CI, confidence intervals*.

*** *p < 0.001*,

** *p < 0.01*,

* *p < 0.05*.

**Table 4:** Stratified variables of stroke patients and binary logistic regression model testing predicting the value of variables with IDDVT.

	**DVT patients**	**Non-DVT patients**	**Unadjusted model**		**Adjusted model 4**	
	**(*N* = 222)**	**(*N* = 222)**	**OR (95% CI)**	***P***	**OR (95% CI)**	***P***
**Age (years)**						
<65, *n* (%)	54 (24.30)	53 (23.90)	1 (ref)		1 (ref)	
≥65, *n* (%)	168 (75.70)	169 (76.10)	0.976 (0.632–1.507)	0.912	0.765(0.461–1.270)	0.3
**Gender**						
Male, *n* (%)	111 (50.00)	150 (67.60)^a^	1 (ref)		1 (ref)	
Female, *n* (%)	111 (50.00)	72 (32.43)^a^	2.083 (1.418–3.062)	<0.001^***^	1.413 (0.878–2.273)	0.155
**Body temperature (** **°** **C)**						
<38°C, *n* (%)	209 (94.1)	217 (97.7)	1 (ref)		1 (ref)	
≥38°C, *n* (%)	13 (5.9)	5 (2.3)	2.700 (0.946–7.705)	0.063	2.289 (0.723–7.252)	0.159
**Stroke type**						
Ischemic stroke, *n* (%)	149 (67.10)	142 (64.00)	1 (ref)		1 (ref)	
Intracerebral hemorrhage, *n* (%)	73 (32.90)	80 (36.00)	0.870 (0.588–1.287)	0.485	1.493 (0.805–2.768)	0.203
**Infection**						
Yes, *n* (%)	45 (20.30)	26 (11.70)^a^	1.917 (1.135–3.236)	0.015^*^	1.485 (0.807–2.736)	0.204
No, *n* (%)	177 (79.70)	196 (88.30)^a^	1 (ref)		1 (ref)	
**NIHSS score at admission**						
<11, *n* (%)	175 (78.8)	177 (79.70)	1 (ref)		1 (ref)	
≥11, *n* (%)	47 (21.20)	45 (20.30)	1.056 (0.667–1.672)	0.815	0.762 (0.433–1.343)	0.347
**Lower limb NIHSS score**						
<2, *n* (%)	105 (47.30)	128 (57.70)^a^	1 (ref)		1 (ref)	
≥2, *n* (%)	117 (57.70)	94 (42.30)^a^	1.517 (1.043–2.207)	0.029^*^	1.246 (0.782–1.984)	0.355
**Padua score**						
<4, *n* (%)	31 (14.00)	56 (25.20)^a^	1 (ref)		1 (ref)	
≥4, *n* (%)	191 (86.00)	166 (74.80)^a^	2.079 (1.279–3.378)	0.003^**^	2.704 (1.261–5.796)	0.011^*^
**RBC (×10** ^**1**^ **2/L)**						
<4.375, *n* (%)	124 (55.90)	107 (48.20)	1 (ref)		1 (ref)	
≥4.375, *n* (%)	98 (44.10)	115 (51.80)	0.735 (0.506–1.068)	0.107	1.229 (0.686–2.202)	0.488
**WBC (×10** ^**9**^ **/L)**						
<10, *n* (%)	190 (85.60)	185 (83.30)	1 (ref)		1 (ref)	
≥10, *n* (%)	32 (14.40)	37 (16.70)	0.842 (0.503–1.409)	0.513	0.702 (0.385–1.281)	0.249
**H.B. (g/L)**						
<135, *n* (%)	103 (63.50)	141 (46.50)^a^	1 (ref)		1 (ref)	
≥135, *n* (%)	119 (53.60)	81 (36.50)^a^	0.497 (0.340–0.727)	<0.001^***^	0.598 (0.324–1.103)	0.100
**PLT (×10** ^**9**^ **/L)**						
<213, *n* (%)	119 (53.60)	124 (55.90)	1 (ref)		1 (ref)	
≥213, *n* (%)	103 (46.40)	98 (44.10)	1.095 (0.75401.592)	0.634	0.957 (0.628–1.458)	0.837
**PT (s)**						
<13.750, *n* (%)	124 (55.9)	98 (44.1)^a^	1 (ref)		1 (ref)	
≥13.750, *n* (%)	98 (44.1)	124 (55.9)^a^	0.657 (0.448–0.966)	0.014^*^	0.608 (0.389–0.951)	0.029^*^
**APTT(s)**						
<36.600, *n* (%)	110 (49.5)	106 (47.7)	1 (ref)		1 (ref)	
≥36.600, *n* (%)	112 (50.5)	116 (52.3)	0.912 (0.625–1.329)	0.631	0.926 (0.600–1.429)	0.728
**FIB (g/L)**						
<4, *n* (%)	143 (64.40)	174 (78.40)^a^	1 (ref)		1 (ref)	
≥4, *n* (%)	79 (35.6)	48 (21.60)^a^		0.001^**^	1.670 (1.027–2.713)	0.039^*^
**D–dimmer (mg/L)**						
<0.5, *n* (%)	24 (10.80)	51 (23.00)^a^	1 (ref)		1 (ref)	
≥ 0.5, *n* (%)	198 (89.20)	171 (77.00)^a^	2.461 (1.453–4.166)	0.001^**^	2.241 (1.242–4.045)	0.007^**^
**Vitamin D status**				0.016^*^		0.033^*^
Sufficiency, *n* (%)	47 (21.2)	73 (32.9)^a^	0.504 (0.315–0.806)	0.004^**^	0.507 (0.300–0.858)	0.011^*^
Insufficiency, *n* (%)	101 (45.5)	79 (35.6)^a^	1 (ref)		1 (ref)	
Deficiency, *n* (%)	74 (33.3)	70 (31.5)	0.827 (0.533–1.284)	0.397	0.686 (0.424–1.111)	0.126

a *indicates statistical difference between DVT and Non-DVT patients (Bonferroni correction was used if multiple comparisons)*.

*DVT, deep vein thrombosis; WBC, white blood cell count; RBC, red blood cell count; HB, hemoglobin value; PLT, platelet count; PT, Prothrombin Time; APTT, activated partial thromboplastin time; FIB, fibrinogen; NIHSS, National Institute of Health stroke scale; OR, odds ratios; CI, confidence intervals*.

*** *p < 0.001*,

** *p < 0.01*,

* *p < 0.05*.

**Table 5:** Multivariate logistic model of DVT among stroke patients.

**Variables**	**β**	**OR (95% CI)**	***P*-value**
Padua score (≥4)	1.001	2.704 (1.261–5.796)	0.011^*^
PT (≥13.75 s)	−0.489	0.608 (0.389–0.951)	0.029^*^
FIB (≥4 g/L)	0.524	1.670 (1.027–2.713)	0.039^*^
D-dimmer (≥0.5 mg/L)	0.804	2.241 (1.242–4.045)	0.007^**^
Vitamin D status			0.033^*^
Sufficiency	−0.655	0.507 (0.300–0.858)	0.011^*^
Insufficiency	Reference	1	Reference
Deficiency	−0.372	0.686 (0.424–1.111)	0.126

*DVT, deep vein thrombosis; WBC, white blood cell count; RBC, red blood cell count; HB, hemoglobin value; PLT, platelet count; PT, Prothrombin Time; APTT, activated partial thromboplastin time; FIB, fibrinogen; NIHSS, National Institute of Health stroke scale; β, regression coefficient; OR, odds ratios; CI, confidence intervals*.

** *p < 0.01*,

* *p < 0.05*.

## Discussion

As far as we know, this was the first research to explore the three-way association among gender, vitamin D, and DVT. There were three main findings in this study. Firstly, females had lower vitamin D levels and a higher risk of DVT than males. Secondly, higher vitamin D levels were associated with a lower risk of DVT. Thirdly, vitamin D may act as a mediating role between gender and DVT.

Accumulating studies had found that elderly females had a higher risk of DVT than males (4), which was in line with our research. In general populations, risk factors such as pregnancy or estrogen antagonist therapy may account for the gender difference in DVT (14), but these factors were not common among elder stroke patients (17). The present study observed that vitamin D might play an essential role between gender and DVT.

Previous research has shown that vitamin D deficiency was more common in elderly, middle-aged, and perimenopausal females (24, 25, 33). According to a retrospective cohort study, as high as 43.9% of elderly females had vitamin D insufficiency (34). Decreased serum levels of vitamin D led to reduced calcium absorption and osteoporosis, which is prevalent among perimenopausal women (33). The previous study has reported the gender difference in dietary intakes of vitamin D. Women tended to intake less amount of vitamin D in wintertime and autumn in a nationwide nutritional survey (26). In an animal experiment, adult ovariectomized female rats had lower recovery levels of 1.25 (OH)_2_ D after 25 (OH) D administration than intact ovary rats, and beta-estradiol administration could restore this decline (28), indicating the critical role estrogen plays in vitamin D transformation. In China, females were more reluctant to be exposed to sunshine and participate in outdoor activities (35), which may be one of the primary causes of hypovitaminosis D. In addition, the gender difference in the amount of body fat may be another possible cause. A previous study demonstrated that vitamin D deficiency was associated with a higher percentage of fat mass in females (36). Given above, the following reasons may partially explain the high prevalence of vitamin D deficiency in elderly females: difference in dietary intake, estrogen deficiency, lower sunlight exposure, and the higher amount of fat in females.

This study also found that vitamin D played a protective role in DVT among stroke patients. The potential role of vitamin D in thrombolytic diseases has been reported formerly. The possible mechanism was that Vitamin D might exert an anti-inflammatory effect through IL-10 receptor expression induction and suppression of NF-κB activation (37, 38) and alleviate oxidative stress in endothelial cells (39) to reduce the chance of blood clots. Besides, it was also worth noting that vitamin D and its analogs may have antithrombotic properties (40, 41) and was reported to modulate coagulation pathways directly or indirectly (42). A pilot randomized clinical trial on DVT and PE patients with vitamin D deficiency found that warfarin's anticoagulation efficacy was enhanced after vitamin D supplement (43). Evidence from a cell study suggested that in monocytic cells, vitamin D inhibited coagulation by downregulating tissue factor (TF), upgrading thrombomodulin (TM), reducing the effects of tumor necrosis factor, and oxidized low-density lipoprotein to exert the antithrombotic activity (44). Other mechanisms of anticoagulation were as follows: modulation of plasminogen activator inhibitor-1 (PAI-1) and thrombospondin-1 expression in smooth cells (45), downregulation, and modulation of coagulation indicators (highly sensitivity c-reactive protein, tissue factor pathway inhibitor, and TNF-α) (46–48).

Hierarchical regression analysis to examine the role of vitamin D in gender and DVT indicated that females had a higher risk of DVT in the univariate model but tended to be more insignificant after multiple adjustments, especially when vitamin D was added. These results showed that vitamin D had a higher statistical power, which could also be interpreted that vitamin D might act as a mediating factor between DVT and the female gender. We speculated that elderly females had lower levels of vitamin D and thus were more likely to develop DVT after stroke. The underlying mechanism might be the decreased properties in anti-inflammation and anti-thromboembolism due to vitamin D insufficiency/deficiency.

Interestingly, our data did not show that vitamin D deficiency could promote DVT after stroke. Current research on the link between vitamin D and VTE has generated inconsistent results. Several studies found that there was no relationship between VTE and Vitamin D deficiency. One study examined the effect of categorized variables of vitamin D (<44 nmol/l or <30 nmol/l) and failed to show a significantly higher risk of VTE (49). Another prospective study over two decades of follow-up also concluded similar results (50). Whereas, other studies reported the lower levels of vitamin D were correlated with DVT or VTE (23, 51, 52), and a pooled HR estimate of 1.25 (95% CI 1.07–1.45) in a Meta-analysis was calculated (50). The inconsistency could be attributed to the difference in study design, race, sample size, latitude, season, criteria of vitamin D deficiency, and dietary habits. In this study, the undefined correlation between vitamin D deficiency and DVT might be due to the limited sample size and cross-sectional design. Further studies including a larger sample, prospective design, and more covariates (such as season) were needed to explore the relationship between vitamin D deficiency and DVT.

There were some limitations in this study. Firstly, as mentioned above, this cross-sectional study could not draw a causal relationship among gender, vitamin D, and DVT. Secondly, we did not record the fat mass and measured the estrogen levels, which may help us justify the relationship between vitamin D levels and elderly females. Thirdly, we only measured the vitamin D concentration once at admission, and we could not observe its fluctuations in stroke patients during hospitalization. Finally, we have not collected the dietary habits and duration of sunlight exposure of the patients daily, which may influence the vitamin D levels.

In conclusion, females had a higher risk of DVT than males among stroke patients, and vitamin D may play an essential role in this relationship. Further studies are needed to explore whether vitamin D supplementation could reduce DVT risk in stroke patients, especially females.

## Data Availability Statement

The raw data supporting the conclusions of this article will be made available by the authors, without undue reservation.

## Ethics Statement

The studies involving human participants were reviewed and approved by Medical Ethics Committee of First Affiliated Hospital of Wenzhou Medical University. Written informed consent for participation was not required for this study in accordance with the national legislation and the institutional requirements.

## Author Contributions

JT: conceptualization, methodology, and formal analysis. FL: data curation and writing—original draft preparation. YL: writing—reviewing and editing and supervision. All authors contributed to the article and approved the submitted version.

## Conflict of Interest

The authors declare that the research was conducted in the absence of any commercial or financial relationships that could be construed as a potential conflict of interest.

## Publisher's Note

All claims expressed in this article are solely those of the authors and do not necessarily represent those of their affiliated organizations, or those of the publisher, the editors and the reviewers. Any product that may be evaluated in this article, or claim that may be made by its manufacturer, is not guaranteed or endorsed by the publisher.
